# Optogenetic control of gut movements reveals peristaltic wave-mediated induction of cloacal contractions and reactivation of impaired gut motility

**DOI:** 10.3389/fphys.2023.1175951

**Published:** 2023-05-15

**Authors:** Yuuki Shikaya, Masafumi Inaba, Ryosuke Tadokoro, Shota Utsunomiya, Yoshiko Takahashi

**Affiliations:** Department of Zoology, Graduate School of Science, Kyoto University Kitashirakawa, Sakyo-ku, Kyoto

**Keywords:** optogeneitcs, gut, peristalsis, chickens, cloaca

## Abstract

Gut peristalsis, recognized as a wave-like progression along the anterior-posterior gut axis, plays a pivotal role in the transportation, digestion, and absorption of ingested materials. The embryonic gut, which has not experienced ingested materials, undergoes peristalsis offering a powerful model for studying the intrinsic mechanisms underlying the gut motility. It has previously been shown in chicken embryos that acute contractions of the cloaca (an anus-like structure) located at the posterior end of the hindgut are tightly coupled with the arrival of hindgut-derived waves. To further scrutinize the interactions between hindgut and cloaca, we here developed an optogenetic method that produced artificial waves in the hindgut. A variant form of channelrhodopsin-2 (ChR2(D156C)), permitting extremely large photocurrents, was expressed in the muscle component of the hindgut of chicken embryos using Tol2-mediated gene transfer and *in ovo* electroporation techniques. The D156C-expressing hindgut responded efficiently to local pulses of blue light: local contractions emerge at an ectopic site in the hindgut, which were followed by peristaltic waves that reached to the endpoint of the hindgut. Markedly, the arrival of the optogenetically induced waves caused concomitant contractions of the cloaca, revealing that the hindgut-cloaca coordination is mediated by signals triggered by peristaltic waves. Moreover, a cloaca undergoing pharmacologically provoked aberrant contractions could respond to pulsed blue light irradiation. Together, the optogenetic technology developed in this study for inducing gut peristalsis paves the way to study the gut movement and also to explore therapeutic methodology for peristaltic disorders.

## Introduction

Gut peristalsis is recognized as a wave-like propagation of a local contraction along the anterior-posterior (A-P; also called oral-aboral) gut axis. The physiology of gut peristalsis has extensively been studied in adults, and the importance of peristaltic movements for the transportation, digestion, and absorption of ingested materials has greatly been appreciated (Huizinga et al., 1998; Huizinga and Lammers, 2009; Spencer et al., 2016). Although it is known that many gut-related disorders are associated with dysfunction of gut motility/peristalsis, efficient therapeutic methods have not been developed. A main reason is that the intrinsic mechanisms underlying the initiation and maintenance of gut peristalsis remain largely unexplored.

It has been shown that the embryonic gut in vertebrates undergoes peristalsis, offering a powerful model to understand the intrinsic mechanisms by which the peristalsis is regulated (Holmberg et al., 2007; Roberts et al., 2010; Chevalier et al., 2017; Chevalier et al., 2019; Chevalier et al., 2020; Okamoto and Hatta, 2022; Shikaya et al., 2022). We have recently shown in chickens that the embryonic cloaca, the posterior orifice of the gut, which is important for excretion of the urine-feces complex (Romanoff, 1960; King and McLelland, 1981), undergoes acute contractions occurring concomitantly with the arrival of hindgut-derived waves (Shikaya et al., 2022). Moreover, when the cloaca is isolated from the hindgut, its contractions are abolished implying intimate coordination between cloaca and its adjacent hindgut (Shikaya et al., 2022). To know whether the cloacal contractions are triggered by the arrival of peristaltic waves from the hindgut, it is necessary to experimentally/artificially produce peristalsis in the hindgut and observe if this manipulation causes acute contractions in the cloaca at the timepoint of wave arrival.

To test this hypothesis, we sought an optogenetic approach whereby we could induce artificial peristaltic waves. A commonly used protein for the optogenetics is channelrhodopsin-2 (ChR2) discovered originally in microbes. This protein is a non-selective cation channel that opens in response to blue light (470 nm) leading to a cellular excitation (Nagel et al., 2003). The wild type and several variants of ChR2 have been used to control the gut motility. While wild type ChR2 and its variant H134R (a mutation in histidine-134 to arginine) evoked mouse and zebrafish gut peristalsis when expressed in the enteric nervous system (ENS) (Hibberd et al., 2018; Perez-Medina and Galligan, 2019), they induced only a local contraction with no following peristaltic waves when expressed in the muscle layer of the gut (Vogt et al., 2021; Okamoto and Hatta, 2022).

In this study, we aimed at optogenetic control of gut peristalsis by targeting the muscle layer of developing gut in chicken embryos, since it is known that myogenic function precedes neural ones during development (Holmberg et al., 2007; Roberts et al., 2010; Chevalier et al., 2020; Shikaya et al., 2022). Recently, another variant D156C was reported to exhibit an extremely large photocurrent and a prolonged open-state lifetime (Dawydow et al., 2014). By optimizing conditions for Tol2-mediated gene transfer with the *D156C*-encoding gene and *in ovo* electroporation targeting the muscle layer of hindgut and cloaca, we found that D156C successfully evoked a local contraction and its following peristaltic movement along the A-P axis in the hindgut. Importantly, when the artificially produced peristaltic waves reached the cloaca, they induced acute contractions in this tissue in accordance with the rhythm of blue light irradiation, demonstrating that the hindgut-derived peristaltic waves mediated the coordination between the hindgut and cloaca. Moreover, cloacae with drug-provoked aberrant contractions were able to respond to blue light pulse to implement rhythmic contractions, suggesting a possible therapeutic methodology for peristaltic disorder.

## Materials and methods

### Chicken embryos

Fertilized chicken eggs were obtained from the Yamagishi poultry farms (Wakayama, Japan). Embryos were staged according to embryonic day (E) or the somite number (somite stage; ss). All animal experiments were conducted with the ethical approval of Kyoto University (#202110).

### Constructions of vectors

The pT2AL200R150G vector was provided by Kawakami (2005). pT2A-CAGGS-mCherry-IRES-Neo^r^ was as previously described (Inaba et al., 2019). pTol2b-nac3-gi-ChR2-mCherry, pTol2b-nac3-gi-ChR2(CSDA)-mCherry, pTol2b-nac3-gi-ChR2(D156C)-mCherry were provided by Aramaki and Kondo (2020). pT2A-CAGGS-ChR2(WT)-mCherry-IRES-Neo^r^: ChR2(WT)-mCherry was PCR-amplified from pTol2b-nac3-gi-ChR2-mCherry. The DNA fragment was subcloned into the EcoRI-Bst XI site of pT2A-CAGGS-ChR2(D156C)-mCherry-IRES-Neo^r^ which was removed ChR2(D156C)-mCherry by EcoRI-Bst XI digestion. pT2A-CAGGS-ChR2(CSDA)-mCherry-IRES-Neo^r^, pT2A-CAGGS-ChR2(D156C)-mCherry-IRES-Neo^r^: ChR2(CSDA)-mCherry and ChR2(D156C)-mCherry was PCR-amplified from pTol2b-nac3-gi-ChR2(CSDA)-mCherry and pTol2b-nac3-gi-ChR2(D156C)-mCherry, respectively. The DNA fragments were subcloned into the NotI site of pT2A-CAGGS-mCherry-IRES-Neo^r^ which was removed mCherry by NotⅠ digestion. pCAGGS-T2TP was as previously described (Sato et al., 2007).

### *In ovo* electroporation

The *in ovo* electroporation was performed as previously described with slight modification (Momose et al., 1999; Atsuta et al., 2013). A DNA solution was prepared at 10 μg/μL (pT2A-CAGGS-mCherry-IRES-Neo^r^, pT2A-CAGGS-ChR2(WT)-mCherry-IRES-Neo^r^, pT2A-CAGGS-ChR2(CSDA)-mCherry-IRES-Neo^r^ or pT2A-CAGGS-ChR2(D156C)-mCherry-IRES-Neo^r^: pCAGGS-T2TP: 4% fast green FCF (Wako, CI 42053) = 4: 1: 0.5), and injected into the coelomic cavity of lateral plate mesoderm in E2.5 (23–25 ss) embryos. An electric pulse of 50 V, 0.05 ms, was given, followed by 5 times of pulses of 7 V, 25 ms, with 250 ms intervals (BEX, Pulse generator CUY21EDITⅡ). Fluorescent images were obtained using the Leica MZ10 F microscope with the DS-Ri1 camera or Nikon A1R confocal microscope.

### Immunohistochemistry

A hindgut was dissected from chicken embryo and fixed in 4% (w/v) paraformaldehyde (PFA)/phosphate buffered saline (PBS: 0.14 M NaCl, 2.7 mM KCl, 10 mM Na_2_HPO_4_–12H_2_O, 1.8 mM KH_2_PO_4_) for 10 min at room temperature (RT). The specimen was washed in PBS twice for 5 min each at RT, and embedded in FSC 22 Clear frozen section compound (Leica, 3801480). Cryostat sections of 20 μm were prepared (Thermo Scientific, Cryostar NX70). Following drying on the hotplate at 37°C, the sections were re-fixed in 4% PFA for 5 min at RT. After washing three times in PBS for 5 min each at RT, the sections were incubated with 0.5% blocking reagent (Roche, 1096176)/PBS for 1 h at RT, followed by primary antibodies; 1:300 dilution of anti-αSMA (abcam, ab5694) and 1:300 dilution of Tuj1 (R & D systems, MAB1195) overnight at 4°C. Following three times washing in PBS for 5 min each at RT, they were incubated with Alexa 488-conjugated second antibodies; 1:300 dilution of anti-rabbit IgG (H + L) (donkey; Invitrogen, A21206), 1:300 dilution of anti-mouse IgG2a (goat; Invitrogen, A21131) and 1:2000 dilution of DAPI (Nacalai Tesque, 11034–56) for 1.5 h at RT. After washing three times for 10 min each at RT, the specimens were sealed with Fluoromount (Diagnostic BioSystems). Fluorescent images were obtained using a Nikon A1R confocal microscope.

### Light stimulation apparatus

The blue light was generated by LED (λ = 470 nm, OSB56L5111P). Trains of light pulses (20-ms pulse width, 5 Hz, 2-s train duration, every 1 min) were delivered focally via glass fiberoptic (250 μm diameter; area of direct illumination: 0.987 mm^2^). Pulse timing was controlled by a microcomputer Arduino Uno (arduino.cc). The light power density was approximately 10 μW/mm^2^, which was measured using a power meter (Thorlabs, PM160). All manipulations were conducted under the red light (λ = 590 nm, Opto Code, EX-590 and LED-EXTA).

### Monitoring of gut motility and kymograph preparation

A portion of gut posterior to the duodenum was dissected from E12 embryos, and placed on a silicone-coated Petri dish (6 cm diameter) filled with 10 mL of high-glucose DMEM (Wako, 048–33575) warmed at 38.5°C with a heating plate (MSA Factory, PH200-100/PCC100G). These procedures were performed under the red-light condition when ChR2-expressing guts were analyzed. To avoid drifts of the specimen during imaging, gut-attached remnants such as pancreas, vitelline membrane, and the surrounding tissues of the cloaca were pinned to the silicone dish with fine needles. Following 10 min of resting, time-lapse images were captured using the Leica MZ10 F microscope with a DS-Ri1 camera (Nikon). The obtained images were processed by ImageJ software (NIH) to analyze gut peristalsis and converted to kymographs as previously described (Shikaya et al., 2022). For the experiments demonstrated in Figures 5, 6, the medium containing 100 μM carbenoxolone (nacalai tesque, 32775–51) or water (Merck Millipore, Elix essential UV3) was used.

### Quantification of cloacal contractions

Our region-of-interest (ROI) was set around the cloaca, and changes in the movement of ROI in captured images were converted to intensity values using Stack Difference of ImageJ. The values of intensity were normalized by the first frame of filmed data. Peaks of contractions were detected using SciPy library (scipy.signal.find_peaks) in Python (parameters: height = 2, distance = 10 s, prominence = 1). Intervals were calculated by the time between two successive peaks. Peaks appearing within 6.5 s after photo-stimulation were defined as the light-induced contractions. Responsivity was calculated by the number of light-induced contractions to all photo-stimulation (10 times).

### Statistical analysis

The box plots represent the median, upper and lower interquartile. Wilcoxon rank-sum test was conducted using R to compare data statistically. Graphs were made by R or matplotlib and seaborn library in Python.

## Results

### Genetic labeling of the muscle layer of hindgut and cloaca

**FIGURE 1:**
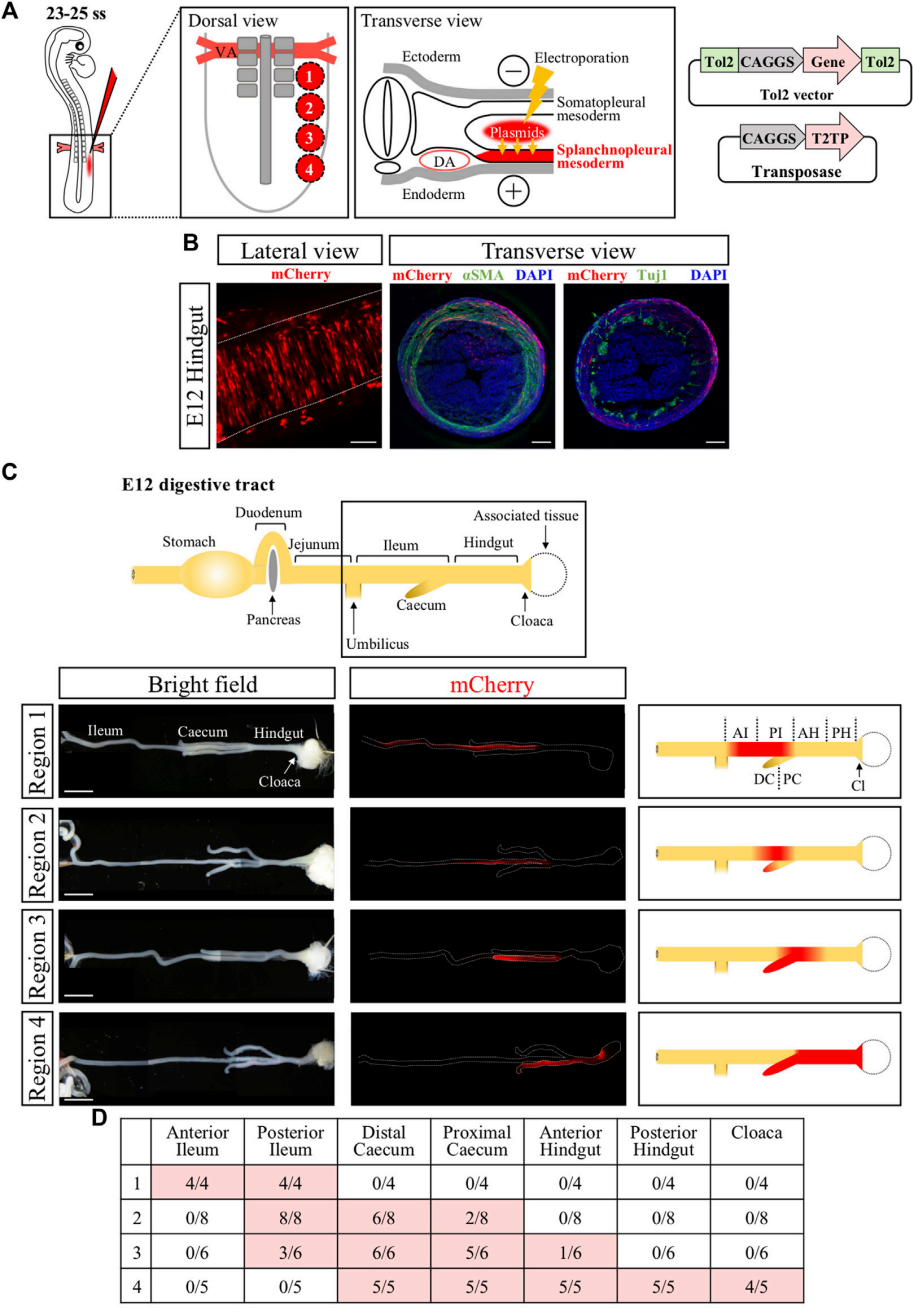
Genetic labeling of hindgut-cloaca muscle layers by *in ovo* electroporation into the splanchnopleural mesoderm. **(A)** The region of splanchnopleural mesoderm in the range from vitelline artery (VA) to the end of the neural tube was divided into 4 regions: region 1, region 2, region 3, and region 4. Each area was roughly targeted by mCherry plasmids by *in ovo* electroporation at 23–25 ss and re-incubated until E12. Tol2-expression vector and transposase are shown. **(B)** mCherry^+^ cells were observed in circular smooth muscles in the gut at E12. Lateral view shows that the mCherry^+^ cells (red) are arranged in the circumferential direction. Transverse view shows immunostaining of αSMA and Tuj1 (green in both). mCherry signals were partially detected in αSMA^+^ cells but not in Tuji1^+^ cells. Nuclei were stained with DAPI (blue). **(C)** A schematic view of the intact gut from duodenum to cloaca. The gut from the ileum to the cloaca (frame) was analyzed. mCherry^+^ cells (red signals in fluorescent views, red color in the schematic diagrams) in regions 1 to 4 are shown in the ileum, posterior ileum and caecum, posterior ileum to anterior hindgut/caecum, and caecum/hindgut to cloaca, respectively. **(D)** Table shows the number of mCherry^+^ gut over the total examined. Scale bars: B, 100 μm; C, 5 mm. ss, somite stage; DA, dorsal aorta; AI, anterior ileum; PI, posterior ileum; DC, distal caecum; PC, proximal caecum; AH, anterior hindgut; PH, posterior hindgut; Cl, Cloaca.

To efficiently express the *ChR2* gene into the muscle layer of developing hindgut and cloaca, we determined the presumptive regions of hindgut and cloaca in the splanchnopleural mesoderm at E2.5 (23ss–25ss) embryos that participates in forming the gut muscle layer (Romanoff, 1960). We divided an area of splanchnopleural mesoderm spanning from the vitelline artery to the posterior end of neural tube into four different regions (#1 to #4) along the A-P axis, and targeted these regions roughly with *CAGGS-mCherry* (Figure 1A). In this study, the gene expression into the splanchnopleural mesoderm was carried out using the Tol2-mediated *in ovo* electroporation (Figure 1A) (Sato et al., 2007; Takahashi et al., 2008; Sato and Takahashi, 2009). In all cases with regions #1 to #4, mCherry-positive cells at E12 were observed in the muscle layer of developing gut (such as circular muscles), consistent with previous studies (Figures 1A,B) (Mahadevan et al., 2014; Sanketi and Kurpios, 2022). Transverse views of electroporated hindgut confirmed that mCherry-positive cells were contained in the muscle layer without overlapping with neural population (neural crest derived) revealed by staining with Tuj1 or αSMA antibodies (Figure 1B, Supplementary Figure S1). We found that the regions #1 to #4 along the A-P axis at E2.5 largely corresponded to labelled regions along the A-P axis at E12 in the gut tube ranging from the ileum to the cloaca (Figure 1C), with the region #4 contributing to the hindgut-cloaca region (Figures 1C, D). In the following experiments, we focused on the region #4 to manipulate the muscle layer of hindgut and cloaca.

### Optogenetic control of gut motility with ChR2(D156C) variant

**FIGURE 2:**
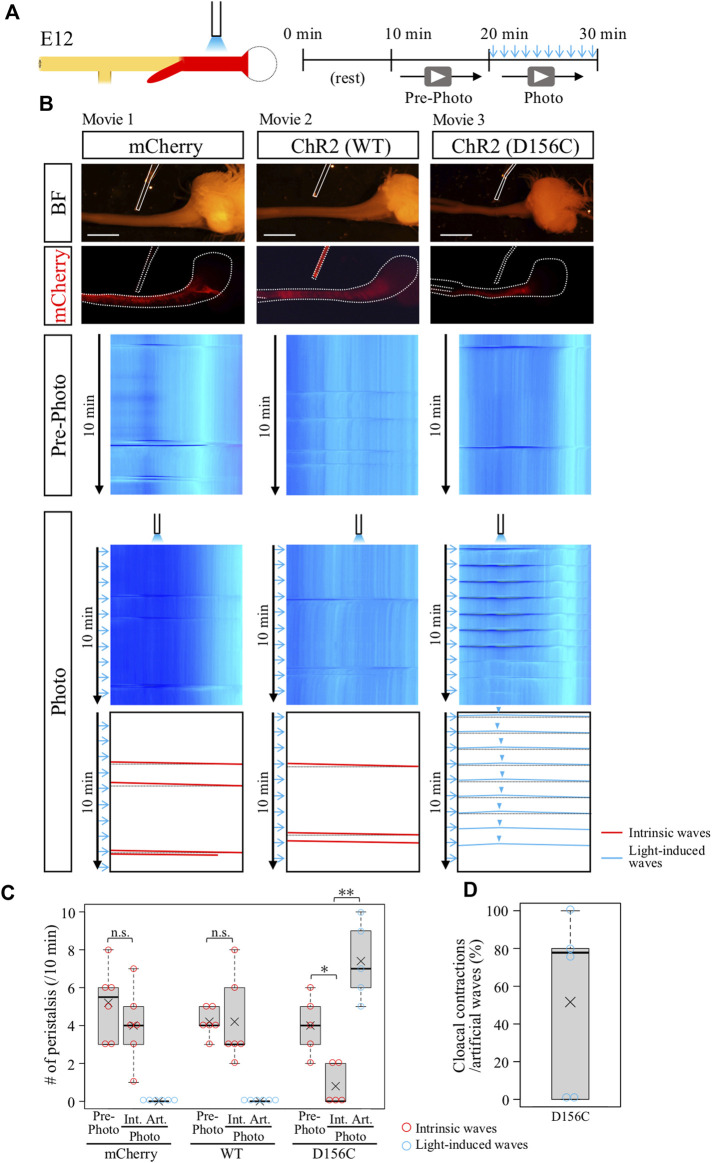
Optogenetic control of hindgut peristaltic waves followed by cloacal contraction. **(A)** Specimen was video recorded for 10 min each before and during photo-stimulation. Blue light irradiation was focally delivered by a fine optical fiber to the middle of hindgut expressing ChR2 (red) with the intermittent stimulation cycle (20-ms pulse width, 5 Hz, 2s, every 1 min). **(B)** DNAs of *mCherry, ChR2 (WT, wild type), or ChR2 (D156C)* were expressed in the muscle layer of hindgut-cloaca by *in ovo* electroporation. White dotted lines and rods indicate the outlines of the gut tube and optical fibers, respectively. Only the intrinsic peristalsis (the red slanted lines) and cloaca contractions (black horizontal lines) were shown in mCherry- and wild-type ChR2-expressing guts in both the pre-photo and the photo recordings (n = 6, n = 6, respectively). In the D156C-expressing guts, while sporadic intrinsic waves were observed in pre-photo recordings, upon the photo-stimulation light-induced waves were evoked propagating both anteriorly and posteriorly represented by inverted V-shaped blue lines in kymograph (n = 5). Origins of the peristaltic waves are indicated by light blue arrowheads. **(C)** Frequency of intrinsic or artificial peristalsis in the 10 min recording. Red and light blue circles represent intrinsic (Int.) and artificial (Art.) waves, respectively. mCherry (Pre-photo, Int., Art.): medians, 5.5, 4, 0/10 min; average (represents “×” hereafter), 5.2, 4.0, 0.0/10 min. WT (Pre-photo, Int., Art.): medians, 4, 3, 0/10 min; average, 4.2, 4.2, 0.0/10 min D156C (Pre-photo, Int., Art.): medians, 4, 0, 7/10 min; average, 4.0, 0.8, 7.4/10 min. **(D)** Cloacal contractions triggered by artificial peristalsis. Median, 77.8%; average, 51.5%. Scale bars, 3 mm; n.s., not significant (*p* > 0.05); *, *p* < 0.05; **, *p* < 0.01.

The ChR2-expressing gut region was dissected from E12 embryos, and subjected to blue light irradiation *ex vivo* (see Materials and Methods). Exploiting the observation that the middle site in the hindgut never exhibits an origin of peristaltic waves [OPWs, (Shikaya et al., 2022)], this site was focally irradiated with blue light using a fine optic fiber at 1-min intervals (Figure 2A). We used the wild-type and D156C variant of ChR2 (Figure 2B). Control hindgut (mCherry-expressed) exhibited no additional OPWs or waves in the hindgut upon irradiation, whereas intrinsic waves normally occurred (Figures 2B,C, red slanted lines; n = 6, Supplementary Movie S1).

Wild-type ChR2 also failed to evoke ectopic contraction/peristalsis (Figures 2B,C, n = 6, Supplementary Movie S2). As previously reported, the frequency of intrinsic waves in the hindgut propagating from the ileum was variable between individuals (Shikaya et al., 2022). The horizontal lines in the kymograph shown in black dotted lines in the trace (Figure 2B) indicate a secondary effect in the hindgut being pulled by acute contractions of the cloaca (Shikaya et al., 2022), which is also explained below.

In clear contrast, the D156C-expressing hindgut responded efficiently to the light irradiation, in which the irradiated site displayed a local contraction in response to blue light that was followed by wave propagation along the hindgut (Figures 2B,C; n = 5, Supplementary Movie S3). Intriguingly, these induced waves were recognized as inverted v-shaped lines in kymograph (blue lines, D156C in Figure 2B), showing that the waves propagated both anteriorly (to stomach) and posteriorly (to cloaca), contrasting with the normal hindgut in which waves propagate only posteriorly. These observations suggest that the hindgut has a potential to accommodate waves in both directions. In addition, the optogenetic induction of artificial waves reduced the occurrence rate of intrinsic peristaltic movements (Figures 2B,C). These phenomena are accounted for by the observation that blue light-induced waves proceeding anteriorly into the ileum, which are seen in Supplementary Movie S3 and highlighted in Supplementary Figure S2, met intrinsic waves coming posteriorly from the ileum resulting annihilation (Chevalier et al., 2017; Chevalier, 2022; Shikaya et al., 2022). We also tested another variant C128S/D156A (CSDA) with an open-state lifetime and photocurrents longer and smaller than those in D156C, respectively (Dawydow et al., 2014), but this variant failed to evoke artificial contraction/waves (data not shown).

### Artificial peristaltic waves caused cloacal contractions

In the same specimens shown in Figure 2 (D156C), in which blue light was irradiated at the middle of hindgut, the reaction of the cloaca was also recorded to examine whether the hindgut-derived waves caused acute contractions of this tissue. As previously reported and explained above, the acute and intense contraction of cloaca pulls the hindgut simultaneously, which is displayed as a horizontal line in kymograph (Shikaya et al., 2022). Importantly, the kymograph of the D156C-hindgut exhibited reiterated horizontal lines (shown in dotted black lines) coupled with the arrival of the optogenetically produced waves from the hindgut (Figures 2B,D, median; 77.8%, average; 51.6%, Supplementary Movie S3), indicating that the hindgut-derived waves caused the acute contractions in cloaca. Together, we concluded that in the developing hindgut, the cloacal acute contractions are mediated by the arrival of hindgut-derived peristaltic waves at least at E12.

**FIGURE 3:**
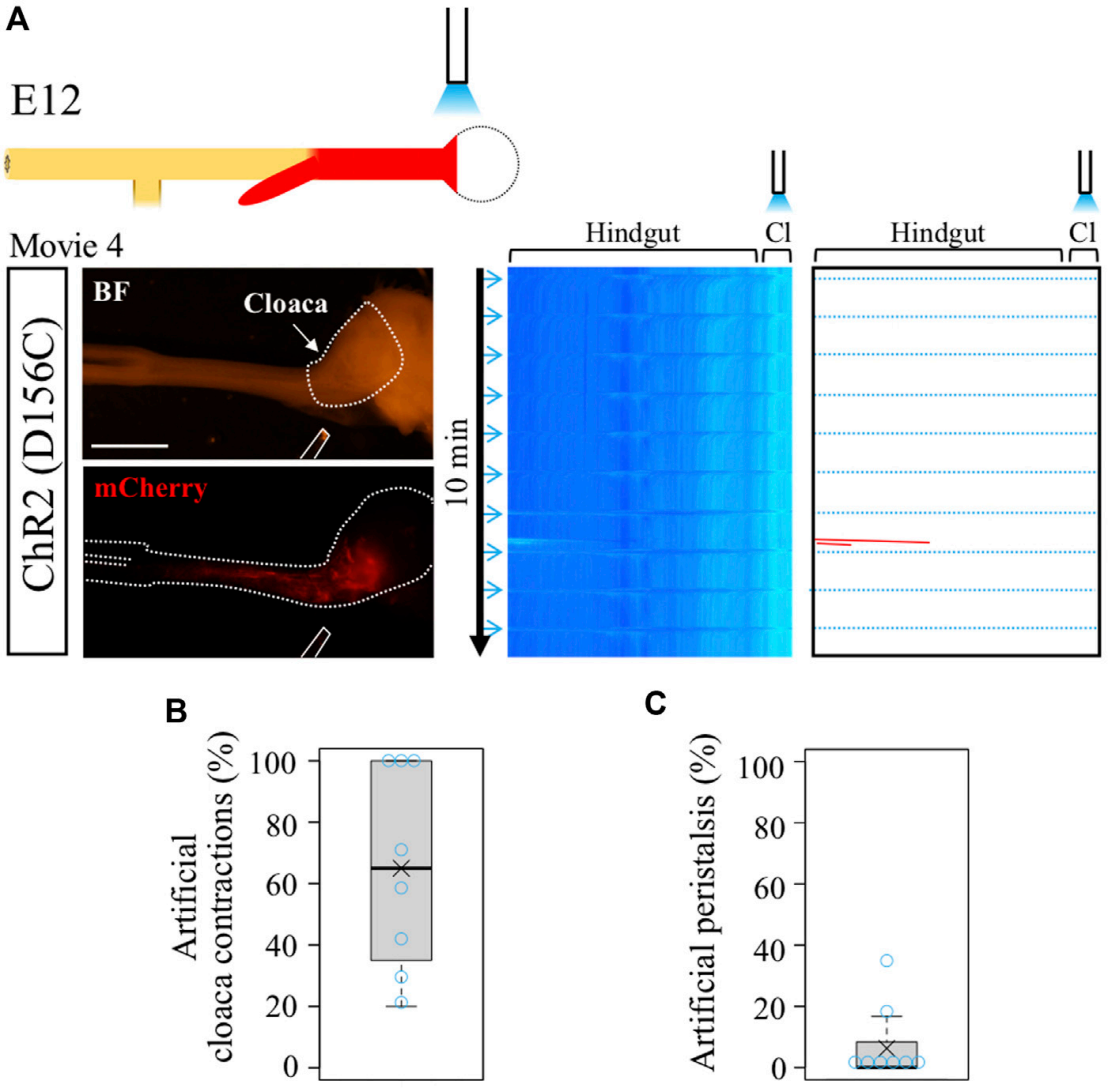
Optogenetic control of cloacal contractions. **(A)** Blue light irradiation of cloaca (shown as white dotted lines in BF image) in the intermittent stimulation cycle as shown in Figure 2. ChR2(D156C)-mCherry was expressed in hindgut-cloaca. White rods indicate optical fibers. Light blue horizontal lines represent artificial cloacal contractions and red slanted lines show intrinsic peristalsis. Artificial cloacal contractions were evoked upon the photo-stimulation (n = 8). **(B)** Percentage of artificial cloacal contractions in all photo-stimulation (10 irradiations in 10 min). Both median and average, 65%. **(C)** Peristalsis in the hindgut evoked by artificial cloacal contractions. Medan, 0%; average, 6.3%. Scale bar, 3 mm.

In the normal hindgut, wave-triggered cloacal contractions do not give impact reciprocally to the hindgut except for the aforementioned pulling effect (recognized as a horizontal line in kymograph). Is this attributed to a refractory period of the hindgut juxtaposed to the cloaca? The optogenetic approach developed in this study allowed us to address this question. Focal irradiation was given to the cloacae in the D156C-expressing guts (Figure 3A), and motility in the hindgut was analyzed by kymography (Figure 3A). We found that blue light successfully induced artificial contractions in the cloacae, which was recognized by horizonal lines (dotted blue lines) in the kymograph (median and average; 65%, Figure 3B, Supplementary Movie S4). However, these cloacal contractions induced very few, if any, additional waves in the hindgut, which would have been detected as leftward slanting lines (Figure 3C, median; 0%, average; 6.3%). Given that the hindgut possesses a potential to accommodate peristalsis in both directions (as revealed in Figure 2), these observations imply that in the normal embryonic gut, the wave-mediated signal is transmitted uni-directionally from the hindgut to the cloaca, for which the refractory period of the hindgut is irrelevant. The uni-directional signaling from hindgut to cloaca revealed in the current study using embryos is reasonable, considering that in adults inter-luminal contents conveyed through the rectum need to be excreted out from the cloaca.

### Contraction-ceased isolated cloacae resumed rhythmic contractions by blue light irradiation

**FIGURE 4:**
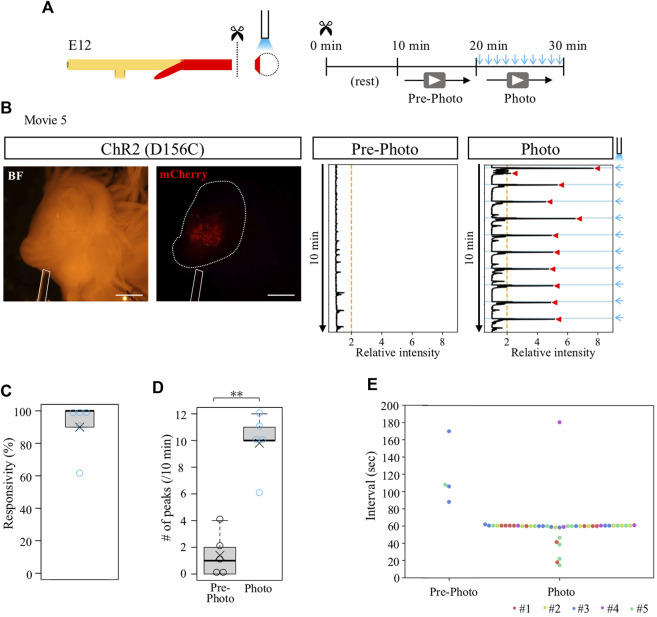
Contraction-ceased isolated cloaca resumed periodic contractions upon photo-stimulation. **(A)** An isolated cloaca was video recorded as shown in Figure 2. **(B)** Expression of ChR2(D156C) in cloaca (white outline) (n = 5). An optic fiber is depicted as a white rod. Orange line represents the threshold (relative intensity 2), the light blue lines indicate the timing of photo-stimulation (1 min intervals), and red arrowheads represent cloacal contractions. **(C)** Responsivity to photo-stimulation in D156C-expressing cloaca. Median, 100%; average, 90%. **(D)** The number of contractions in the cloaca expressing D156C. Black and light blue circles represent intrinsic (Pre-photo) and artificial (Photo) contractions, respectively. Pre-photo: median, 1 peak/10 min; average, 1.4 peaks/10 min; n = 5. Photo: median, 10 peaks/10 min; average, 9.8 peaks/10 min; n = 5. *p*-value was calculated by Wilcoxon rank-sum test. **(E)** Intervals of the contractions in the cloaca expressing D156C. Dots in different colors correspond to different individuals (n = 5, #1-#5). No contractions were detected in the three specimens during pre-photo recordings. Scale bars, 1 mm; **, *p* < 0.01.

We previously reported that the cloaca ceases its contractions when it is separated from the hindgut (Shikaya et al., 2022). To clarify whether the isolated cloaca had lost or retained their contractile potential, we stimulated the isolated cloacae using the optogenetic method developed in this study. From D156C-expressing guts, the cloacae were isolated from the hindguts and subjected to blue light irradiation (Figure 4A). After transferring into a Petri dish, the cloacae were allowed to rest for another 10 min followed by video recordings for 10 min each before (control) and during irradiation (Figure 4A). Relative intensity of contractions was calculated using the Stack Difference of ImageJ as previously reported (Figure 4B) (Shikaya et al., 2022). For quantification, a peak that appeared above the relative intensity 2 within 6.5 s after photo-stimulation was defined as the light-induced peak of contractions. While an isolated D156C-cloaca almost ceased its contraction (median; 1 peak/10 min, average; 1.4 peaks/10 min, n = 5), it was re-activated by pulsed blue light (10 times/10 min) with high responsivity to the irradiation rhythm (median; 10 peaks/10 min, average; 9.8 peaks/10 min) (Figures 4B–D, Supplementary Movie S5). Responsivity was calculated by the number of light-induced contractions to photo-stimulation (10 times). Accordingly, intervals for the repeated peaks in the irradiated cloaca were predominantly around 60 s (Figure 4E; 5 specimens were shown in different colors). The isolated cloaca therefore retains its contractile potential when removed from the rest of the gut.

### Isolated D156C-cloaca with drug-provoked aberrant contractions was able to respond to pulsed irradiation

**FIGURE 5:**
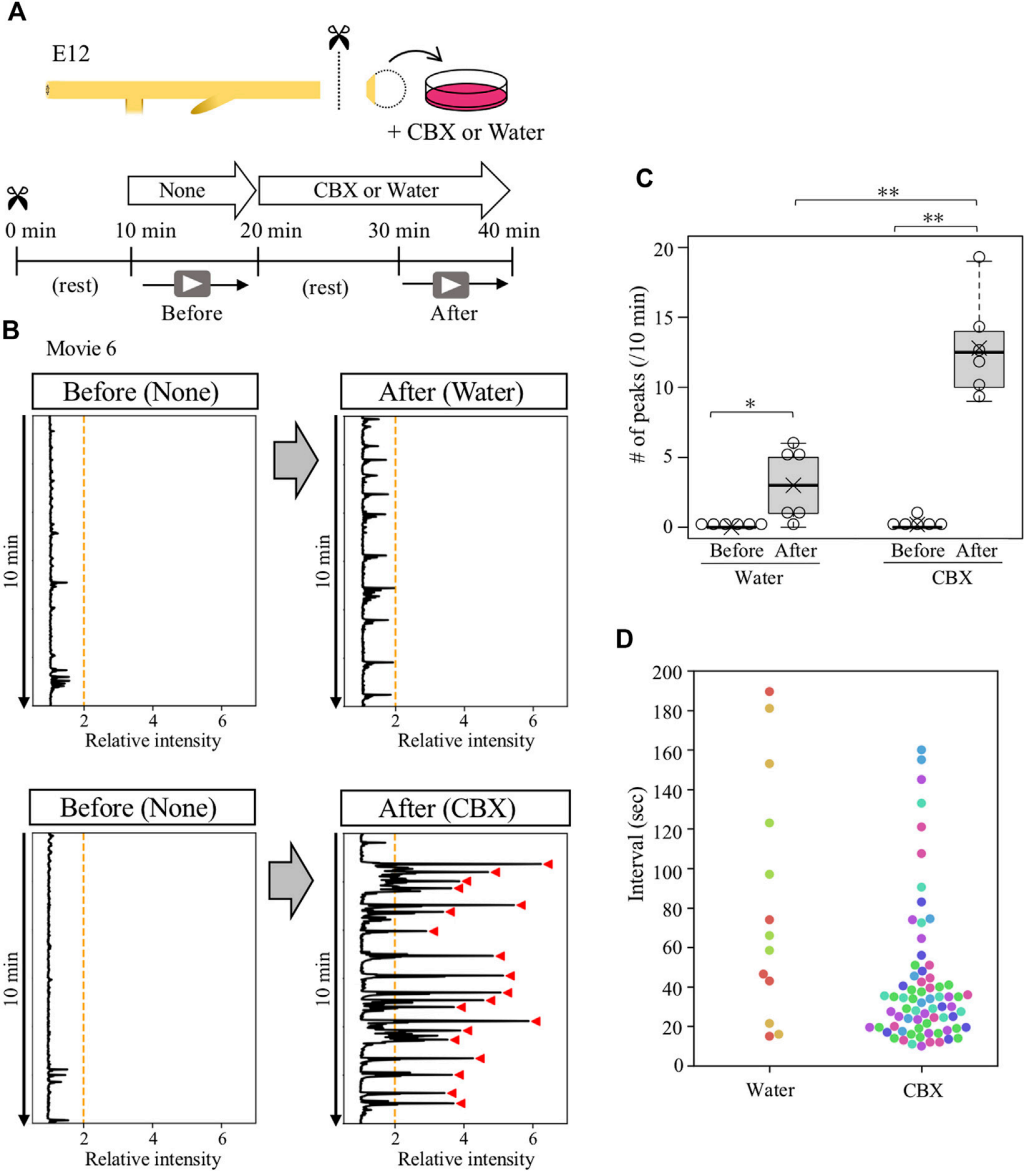
Gap junction inhibitor CBX caused abnormal contractions in contraction-ceased isolated cloaca. **(A)** Cloaca was dissected from the gut and subjected to video recording for 10 min in normal medium, followed by a 10 min rest and second recording in water (control)- or CBX-containing medium. **(B)** Relative intensity of cloacal contractions (n = 6). Orange lines and red arrowhead are as shown in Figure 4. **(C)** The number of contractions during 10 min-recording in water- or CBX-treated cloaca. Water (before, after): medians, 0, 3/10 min; averages, 0, 3/10 min. CBX (before, after): medians, 0, 12.5/10 min, averages, 0.2, 12.8/10 min. *p* values were calculated by Wilcoxon rank-sum test. **(D)** Intervals of the contractions in the water- or CBX-treated cloaca. Dots in different colors correspond to different individuals (n = 6). No contractions were detected in three specimens treated by water. *, *p* < 0.05; **, *p* < 0.01.

To determine whether the ceased- or reactivated contractions of isolated cloaca were mediated by gap junction signals, the isolated cloacae were treated with carbenoxolone (CBX), a drug widely used to inhibit gap junction function, followed by video-recording for 10 min (Figure 5A). The specimen was allowed to rest for 10 min after administration with CBX to avoid possible effects by culture medium turbulence. We found that the CBX-treated cloaca exhibited active but irregular contractions with intense amplitudes compared with control (water-treated) cloacae (Figures 5B,C; median; 12.5 peaks/10 min, average; 12.8 peaks/10 min, Supplementary Movie S6). Intervals of these contraction cycles in the CBX-treated cloacae were random (Figures 5B,D). These observations raised a possibility that in the normal gut, the cloaca possesses a latent ability to undergo spontaneous contractions, which are suppressed by gap junction-mediated signals, and this suppression is temporarily released at the time of the arrival of hindgut-derived waves.

**FIGURE 6:**
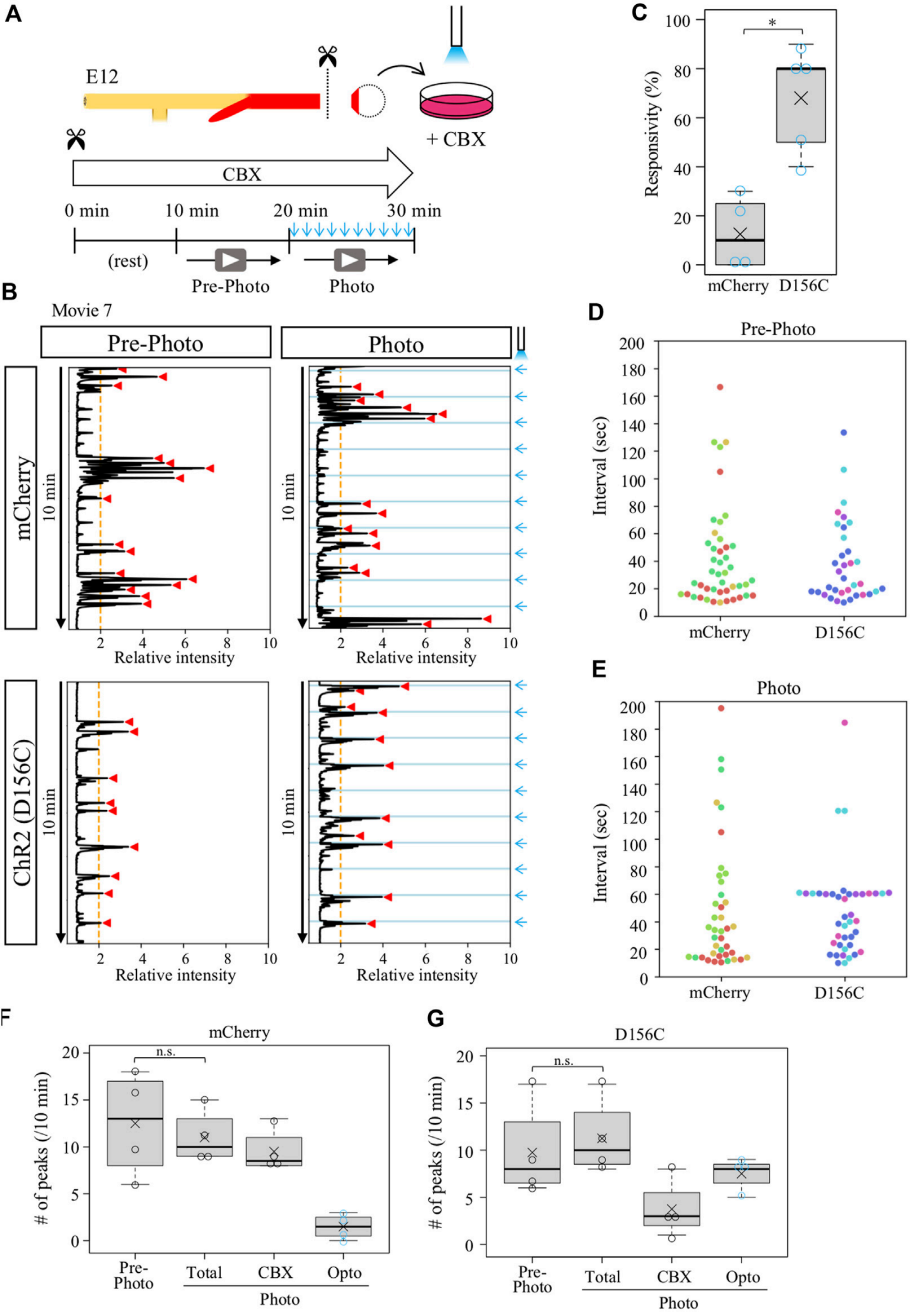
Isolated cloaca with CBX-provoked aberrant contractions could respond to photo-stimulation. **(A)** Recordings were as shown in Figures 2, 4. **(B)** Relative intensity of contractions in the cloaca expressing mCherry or ChR2(D156C) (n = 4 and n = 4, respectively). **(C)** Responsivity to the photo-stimulation. A peak that appeared within 6.5 s after photo-stimulation was defined as light-induced contraction. mCherry: medians, 10%; averages, 12.5%. D156C: medians, 80%; average, 75%. *p*-value was calculated by Wilcoxon rank-sum test. *: *p* < 0.05. **(D)** Peak intervals in mCherry- or D156C-expressing cloaca in pre-photo recordings. A 302-s interval found in the mCherry data set was excluded. Dots in different colors correspond to different individuals (n = 4). **(E)** Peak intervals in mCherry- or D156C-expressing cloaca in photo recordings. Dots in different colors reveal different individuals (n = 4). n.s.: not significant (*p* > 0.05), *: *p* < 0.05. **(F,G)** The number of peaks in the mCherry- or D156C-expressing cloaca. Total number of peaks (Total) means the sum of the number of peaks induced by CBX (CBX) and induced by photo-stimulation (Opto). mCherry (Pre-photo, Total, CBX, Opto): medians, 13, 10, 8.5, 1.5/10 min; averages, 12.5, 11, 9.5, 1.5/10 min D156C (Pre-photo, Total, CBX, Opto): medians, 8, 10, 3, 8/10 min; averages, 9.8, 11.3, 3.8, 7.5/10 min. *p* values were calculated by Wilcoxon rank-sum test. n.s.: not significant (*p* > 0.05).

Given that the isolated D156C-cloacae retained a potential to respond to external stimuli (blue light) as shown in Figure 4, we expected that the CBX-provoked aberrant contractions with irregular intervals could be entrained by regular pulse of irradiation. D156C-cloacae were isolated from the hindguts and soaked in the medium containing CBX, and blue light pulses were delivered every 1 min for 10 min as described above (Figure 6A). Contractions were recorded before and during blue light delivery, and compared between mCherry- and D156C-cloacae. Markedly, the D156C-cloacae responded significantly to the rhythm of optogenetic stimuli (Figure 6B, Supplementary Movie S7) with responsivity of 68% in average (median 80%, n = 4) being much higher than control mCherry-cloacae (average; 12.5%, median; 10%, n = 4) (Figure 6C). The number of intervals around 60 s was prominent in the photo-activated D156C-cloacae compared with that of pre-photo D156C-cloacae and photo-activated mCherry (control)-cloacae (Figures 6D,E). The total number of peaks did not change significantly between before and during irradiation in control/mCherry (12.5 and 11 peaks in average, respectively) and D156C-cloacae (9.8 and 11.3 peaks in average, respectively) (Figures 6F,G). With irradiation, 7.5 peaks (average) were evoked in the D156C-cloacae suggesting that CBX-induced random contractions were reduced in number (Figure 6G). Together, the isolated D156C-cloacae with CBX-provoked aberrant contractions could respond to the blue light to exhibit regular contractions.

## Discussion

We have succeeded for the first time to evoke functional peristaltic waves by optogenetically activating the gut muscle layer. The results obtained with this method have indicated that the coordination between the hindgut and cloaca is mediated by the hindgut-derived peristaltic waves. The D156C variant, but not the wild type, of ChR2 efficiently evokes a local contraction and following peristalsis in the hindgut. Our method also allows the isolated cloaca, which would normally cease its acute contractions, to resume contractions with the rhythm of light irradiation. Furthermore, the cloacae undergoing CBX-provoked aberrant contractions can respond to the external stimuli and implement rhythmic contractions with light pulse.

### ChR2(D156C) efficiently evokes gut contraction and peristalsis in the hindgut

ChR2 and its variants have been used mostly in the field of neurosciences. Recently, ChR2-optogenetics has also been applied to study gut peristalsis. While wild type and/or H134R were able to evoke peristalsis when expressed in ENS (Hibberd et al., 2018; Perez-Medina and Galligan, 2019), no report has been provided in which peristalsis was produced by targeting the gut muscle layer. We have successfully produced local contractions and subsequent peristalsis by optogenetically activating muscle layer cells including smooth muscles in the hindgut using the D156C variant known to show extremely large photocurrents (Dawydow et al., 2014). Since D156C-electroporated splanchnopleural cells might also have contributed to interstitial cells of Cajal (ICCs) thought to be a pace maker, the possibility that D156C-optogenetically evoked peristalsis was initiated by ICCs cannot be excluded. Combined with optogenetic activation of ENS, our method should lay the groundwork for understanding the intricate regulatory network between the ENS and smooth muscle layer during gut peristalsis. In chickens, one study was previously reported in which developing motor neurons were optogenetically manipulated using wild type ChR2 (Kastanenka and Landmesser, 2010).

It is unknown why C128S/D156A (CSDA), which is characterized by very long open-state lifetime, is unable to evoke artificial waves in our study (Figure 2) (Berndt et al., 2009; Bamann et al., 2010). This variant is known to be activated by a low light power of 8 μW/mm^2^ (Yizhar et al., 2011b), which is similar to the light density in our optogenetic apparatus (approximately 10 μW/mm^2^). One possible explanation is that the extremely long open-state lifetime of CSDA (τ = 29 min) compared with D156C (τ = 76 s) might negatively affect the excitation of muscle layer cells/smooth muscles (Yizhar et al., 2011a; Dawydow et al., 2014).

### The coordination between the hindgut and cloaca is mediated by the peristaltic waves

When an optogenetically evoked peristaltic wave arrives at the endpoint of the hindgut, it induces an acute contraction in the cloaca. Together with our previous report showing that the cloaca ceases its contractions when isolated from the hindgut (Shikaya et al., 2022), the findings obtained in the current study demonstrate that the cloacal contractions are triggered by the hindgut-derived peristalsis.

When the hindgut-connected (normal) cloaca is optogenetically activated locally, its contractions do not influence the hindgut peristalsis, whereas the hindgut has a potential to accommodate bidirectionally propagating waves revealed in this study (Figure 2B). These observations suggest that in the normal gut, signal transmission upon the wave arrival at the cloaca is uni-directional, which is reasonable considering the directional transportation of the urine-feces complex out from the cloaca during the excretion in adults.

### Gut optogenetics for a possible therapeutic tool

Optogenetic analyses with the isolated cloaca, which normally ceases its contractions, have further provided three novel findings. One is that the isolated cloaca is able to respond to external stimuli. Second, the ceased contractions of the separated cloaca are mediated, at least partly, by gap junction, since CBX causes aberrant contractions reflecting its latent contraction potential. One explanation for the cessation in the isolated CBX-free cloacae is that suppressors of the smooth muscle contractions, such as the nitric oxide known to be synthesized in smooth muscles or ENS (Llewellyn-Smith et al., 1992; Teng et al., 1998), spread through gap junctions, and CBX inhibits this spreading, resulting in spontaneous contractions. Third, even with the CBX-provoked aberrant contractions, the isolated cloacae can artificially be controlled to implement rhythmic contractions in accordance with blue light irradiation. It is conceivable that in the normal gut, the cloaca is restrained from spontaneous contractions by gap junction-mediated signals, and it is likely that this restraint is temporarily relieved when the hindgut-derived wave arrives. How the wave arrival relieves the gap-junction mediated signals and how such signaling temporarily operates have yet to be clarified.

In summary, the optogenetic method optimized for the gut muscle layer in chicken embryos has provided a powerful tool to decipher the mechanisms by which the gut contractions and peristalsis are regulated. The method also offers a means for noninvasive therapeutic control of gut peristalsis in gut motility-impaired patients.

## Data Availability

The original contributions presented in the study are included in the article/Supplementary Material, further inquiries can be directed to the corresponding author.

## Supplementary material

SUPPLEMENTARY FIGURE 1 Molecular characterization of the hindgut labeled by the electroporation of CAGGS mCherry into splanchnopleural mesoderm. **(A)** Transverse images of the mCherry expressing hindgut, co-staining with αSMA, Tuj1, and DAPI. **(B)** A magnified view of the frame in A. Note that most mCherry expression is detectable in αSMA+ layer with no labeling in neighboring Tuj1+ neurons. Scale bars: A, 50 μm; B, 10 μm.

SUPPLEMENTARY FIGURE 2 The artificial peristaltic waves induced by photo-stimulation propagated anteriorly and reached the ileum. **(A)** Images of stimulated region. The bottom panel showed a magnified view of the square in the upper panel. White rods and lines indicate the outlines of optical fibers and the gut tube, respectively. **(B)** Time-lapse images with pseudo-color corresponding to movie 3. White arrowheads indicate the wavefront of contraction and white rods indicate the outlines of the gut tube and optical fibers. Scale bars, 1 mm. Ile, ileum; Cc, caecum; HG, hindgut.

SUPPLEMENTARY MOVIE 1 Only intrinsic peristalsis and cloaca contractions were observed in the mCherry-expressing gut. Time-lapse images were obtained two frames per second before (control) and during irradiation. A white rod indicates outlines of the optical fiber. This video corresponds to mCherry in Figure 2B.

SUPPLEMENTARY MOVIE 2 Only intrinsic peristalsis and the cloacal contractions were observed in the ChR2(WT)-expressing gut. Time-lapse images were obtained two frames per second before (control) and during irradiation. A white rod indicates the outlines of the optical fiber. This video corresponds to ChR2(WT) in Figure 2B.

SUPPLEMENTARY MOVIE 3 Artificial peristalsis which induced cloacal contractions were observed in the ChR2(D156C)-expressing gut. Time-lapse images were obtained two frames per second before (control) and during irradiation. A white rod indicates the outlines of the optical fiber. This video corresponds to ChR2(D156C) in Figure 2C and S2B.

SUPPLEMENTARY MOVIE 4 Artificial contractions in D156C-cloaca were induced by blue light irradiation. Time-lapse images were obtained two frames per second during irradiation. A white rod 678 corresponds to Figure 3A.

SUPPLEMENTARY MOVIE 5 A contraction-ceased isolated cloaca was induced to undergo artificial contractions by photo-stimulation. Time-lapse images were obtained two frames per second before (control) and during irradiation. A white rod indicates the outlines of the optical fiber. This video corresponds to Figure 4B.

SUPPLEMENTARY MOVIE 6 Aberrant contractions in CBX-treated isolated cloaca. Time-lapse images were obtained two frames per second before (None) and after CBX or water administration. This video corresponds to Figure 5B.

SUPPLEMENTARY MOVIE 7 An isolated cloaca with CBX-provoked abnormal contractions could respond to photo-stimulation. Time-lapse images were obtained two frames per second before (control) and during irradiation. A white rod indicates the outlines of the optical fiber. This video corresponds to Figure 6B.
