# Supplementary figures and images for: Optogenetic control of gut movements reveals peristaltic wave-mediated induction of cloacal contractions and reactivation of impaired gut motility
Source: Front Physiol. 2023 May 15;14:1175951. doi: 10.3389/fphys.2023.1175951 (PMC10245550; doi:10.3389/fphys.2023.1175951)

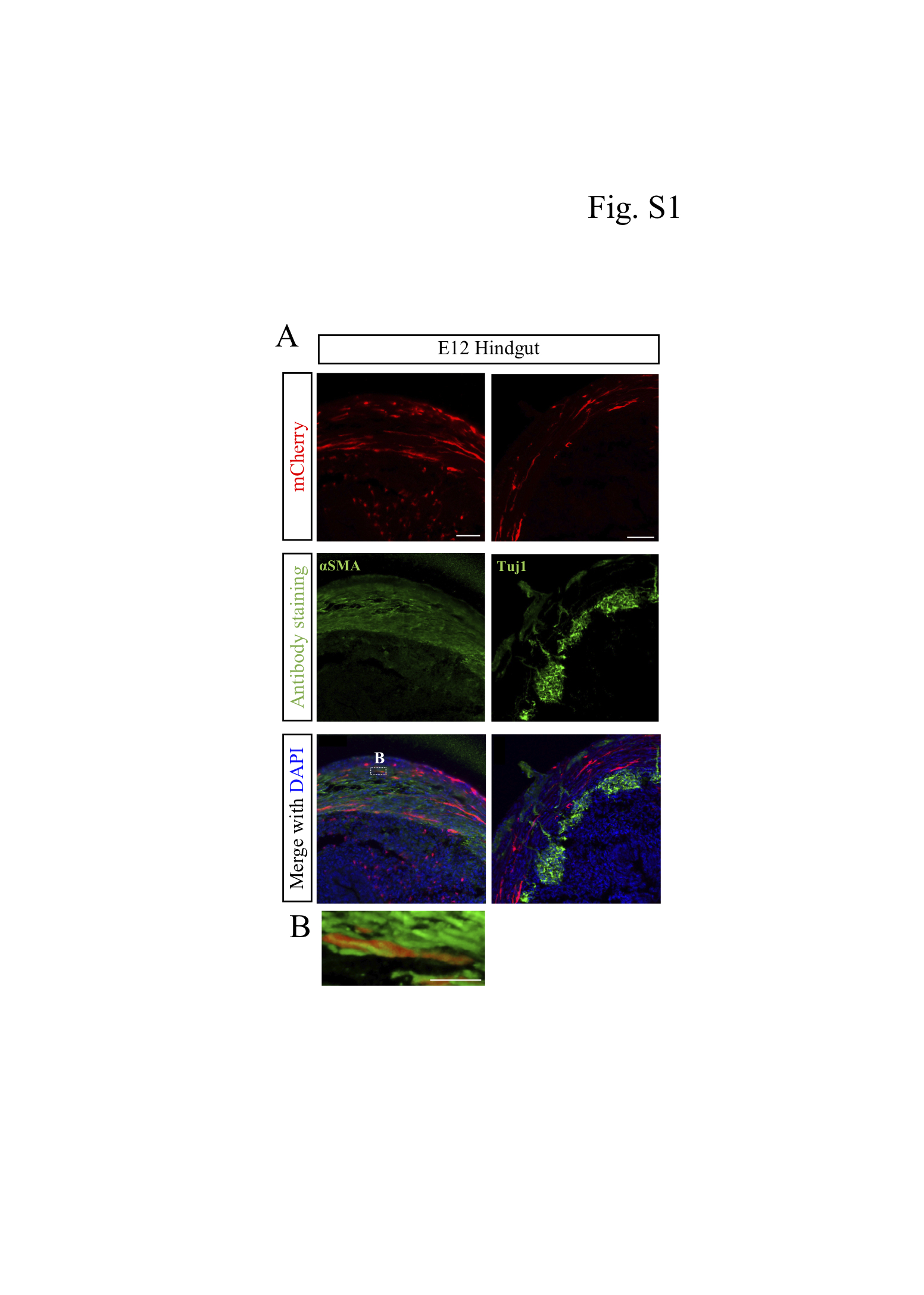

Supplement: Supplementary file 2 [file Image1.jpeg]

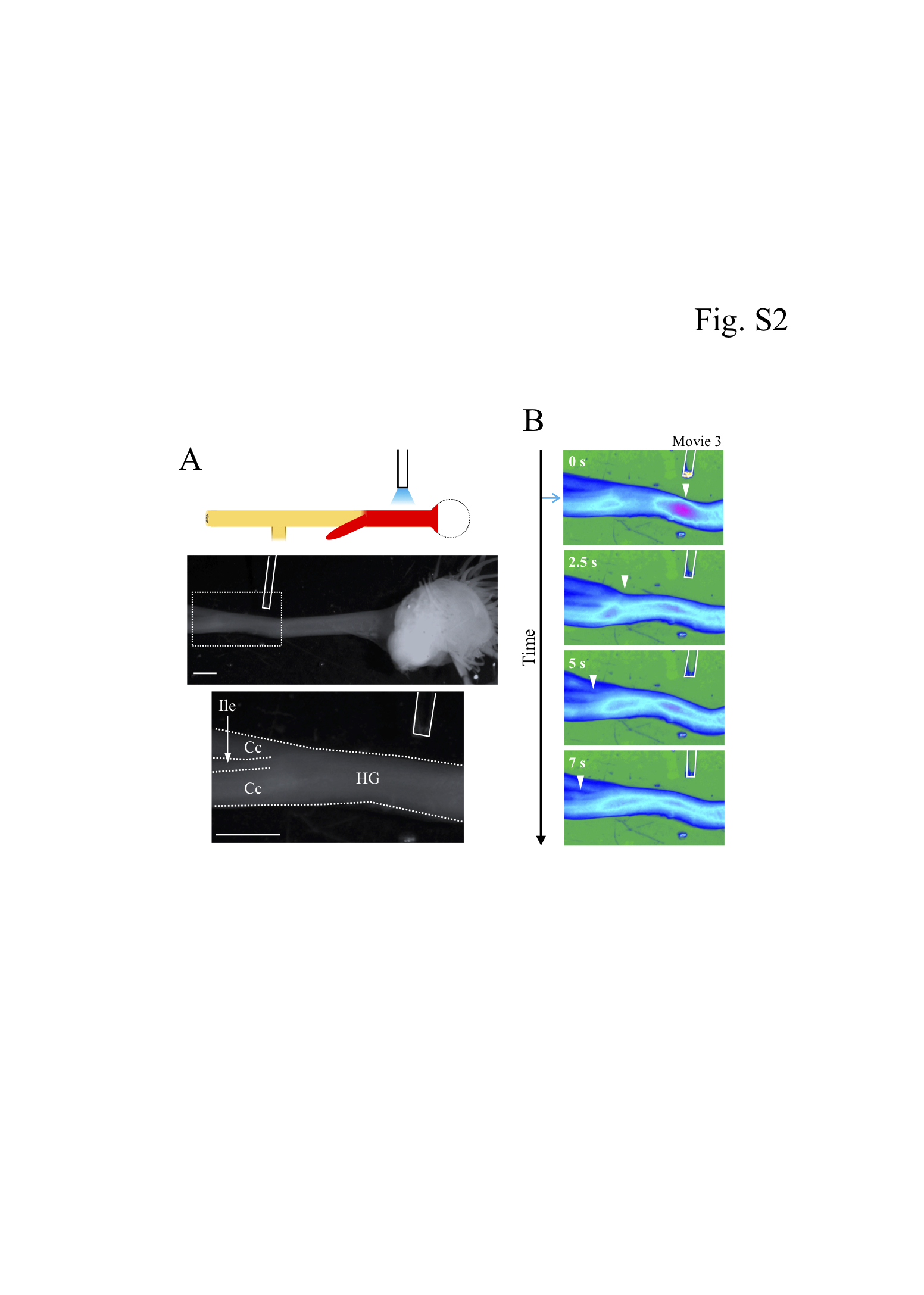

Supplement: Supplementary file 3 [file Image2.jpeg]
